# Supplementary material for: The Rhodanese PspE Converts Thiosulfate to Cellular Sulfane Sulfur in Escherichia coli
Source: Antioxidants (Basel). 2023 May 20;12(5):1127. doi: 10.3390/antiox12051127 (PMC10215317; doi:10.3390/antiox12051127)
Supplement: Supplementary file 1 [file antioxidants-12-01127-s001.zip › antioxidants-2387434-supplementary.pdf]

Supplementary materials for

The rhodanese PspE converts thiosulfate to cellular sulfane sulfur in *E. coli*

Qiaoli Yu[1], Mingxue Ran[1], Yuping Xin[1], Huaiwei Liu[1], Honglei Liu[1], Yongzhen Xia[1*] and Luying Xun[1,2*]

[1] State Key Laboratory of Microbial Technology, Shandong University, 72 Binhai Road, Qingdao 266237, China

[2] School of Molecular Biosciences, Washington State University, Pullman, WA, 991647520, USA

* Corresponding authors: L. Xun. Tel: +1-509-335-2787; Email: luying_xun@vetmed.edu.edu and Y. Xia. Tel: +86-532-58631572; Email: xiayongzhen2002@sdu.edu.cn.

Supplemental Figures S1-7.
Supplemental Tables S1-2.

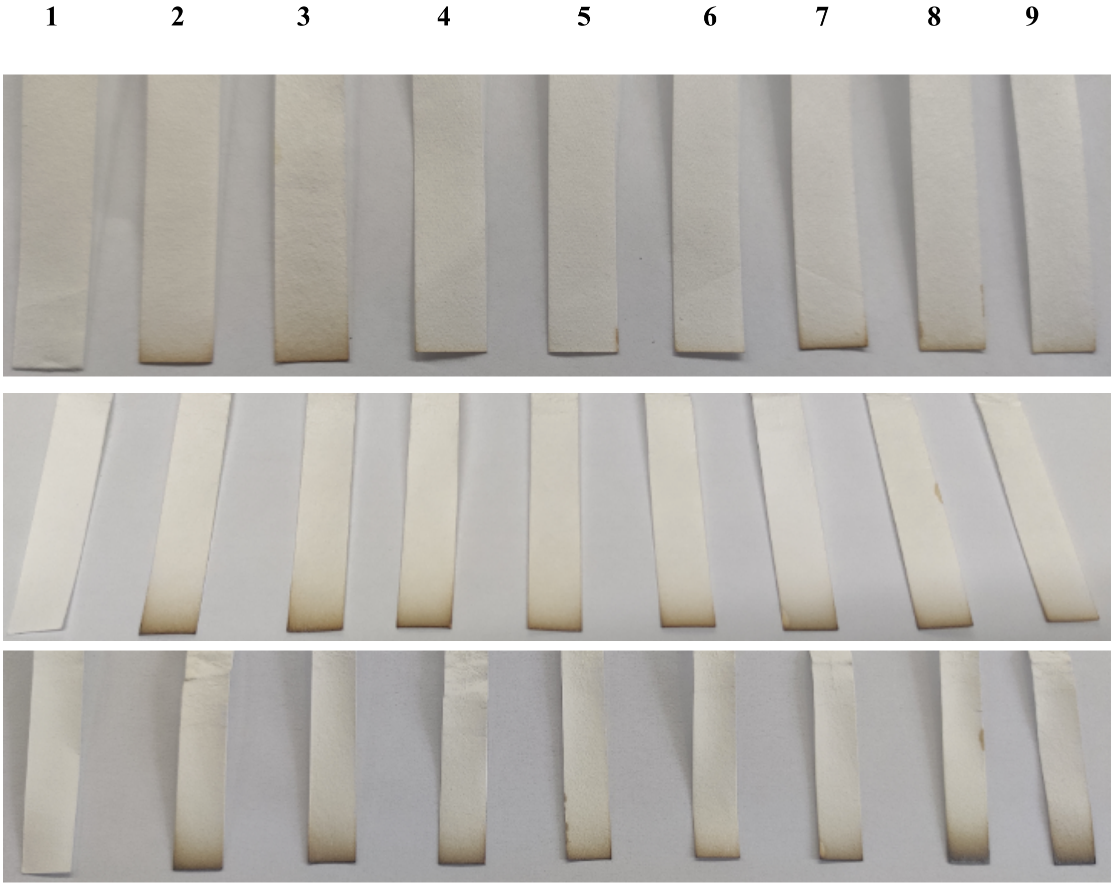

Supplementary Figure S1. Hydrogen sulfide detection after addition of thiosulfate to *E. coli* strains. Lane 1, LB + 10 mM thiosulfate; Lane 2, *E. coli* MG1655 + 0 mM thiosulfate; Lane 3, *E. coli* MG1655 + 10 mM thiosulfate; Lanes 4 - 9, *E. coli* mutants + 10 mM thiosulfate: RHOD-5K (lane 4), RHOD-8K (lane 5), ΔsseA (lane 6), ΔpspE (lane 7), ΔpspE::ΔNS pspE (lane 8), ΔpspE::pspE (lane 9). The cells were cultured in LB till OD_{600nm} of 1, and then thiosulfate and lead-acetate strips were added. The cultures were at 37°C with shaking at 200 rpm. The strips were taken and photographed at 1, 6, and 20 h.

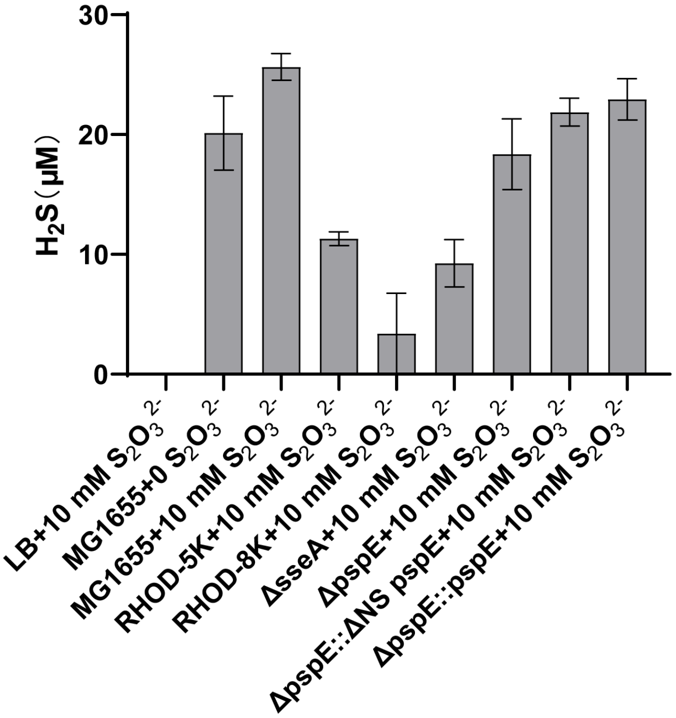

Supplementary Figure S2. Hydrogen sulfide detection by using HPLC after addition of thiosulfate to *E. coli* strains. *E. coli* were transferred to fresh LB at initial OD_{600} = 0.05, and incubated at 37°C, 200 rpm for 30 min, then added 0.4 mM IPTG to induce PspE expression. When the strains grew to OD_{600} of 1, 10 mM thiosulfate was added and incubated at 37°C, 200 rpm for 1 h. One mL bacteria culture of OD_{600} = 2 was taken and centrifuged, and the sulfide in the supernatant was derivatized by mBBr and then detected by HPLC.

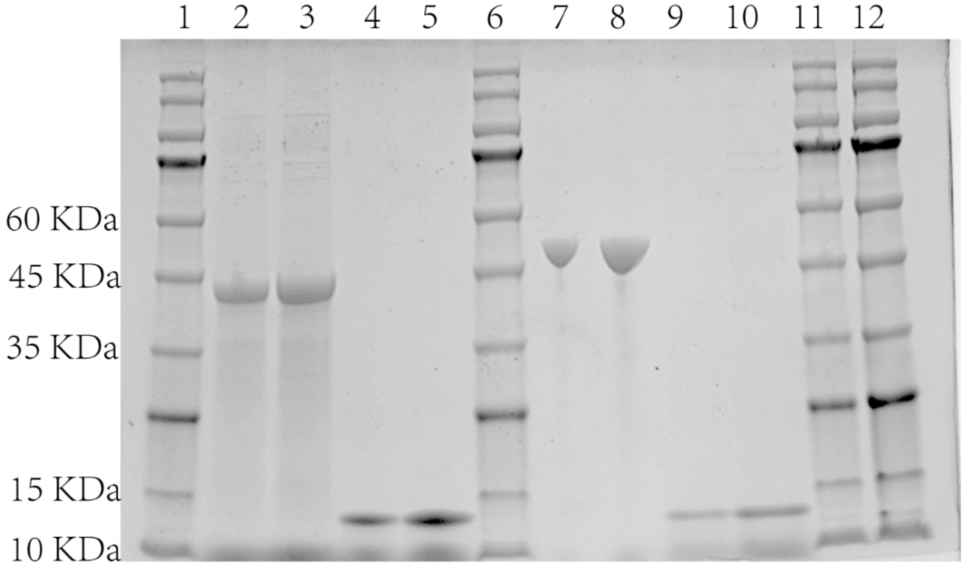

Supplementary Figure S3. SDS-PAGE analysis of purified RHODs. The purified YceA (line 2, 3), ΔNS-PspE (line 4, 5), ΔNS-YnjE (line 7, 8), and GlpE (line 9, 10) were analyzed via SDS-PAGE. The *Blue Plus* IV Protein Marker (10 KDa~180 KDa) was used in lines 1, 6, 11, and 12.

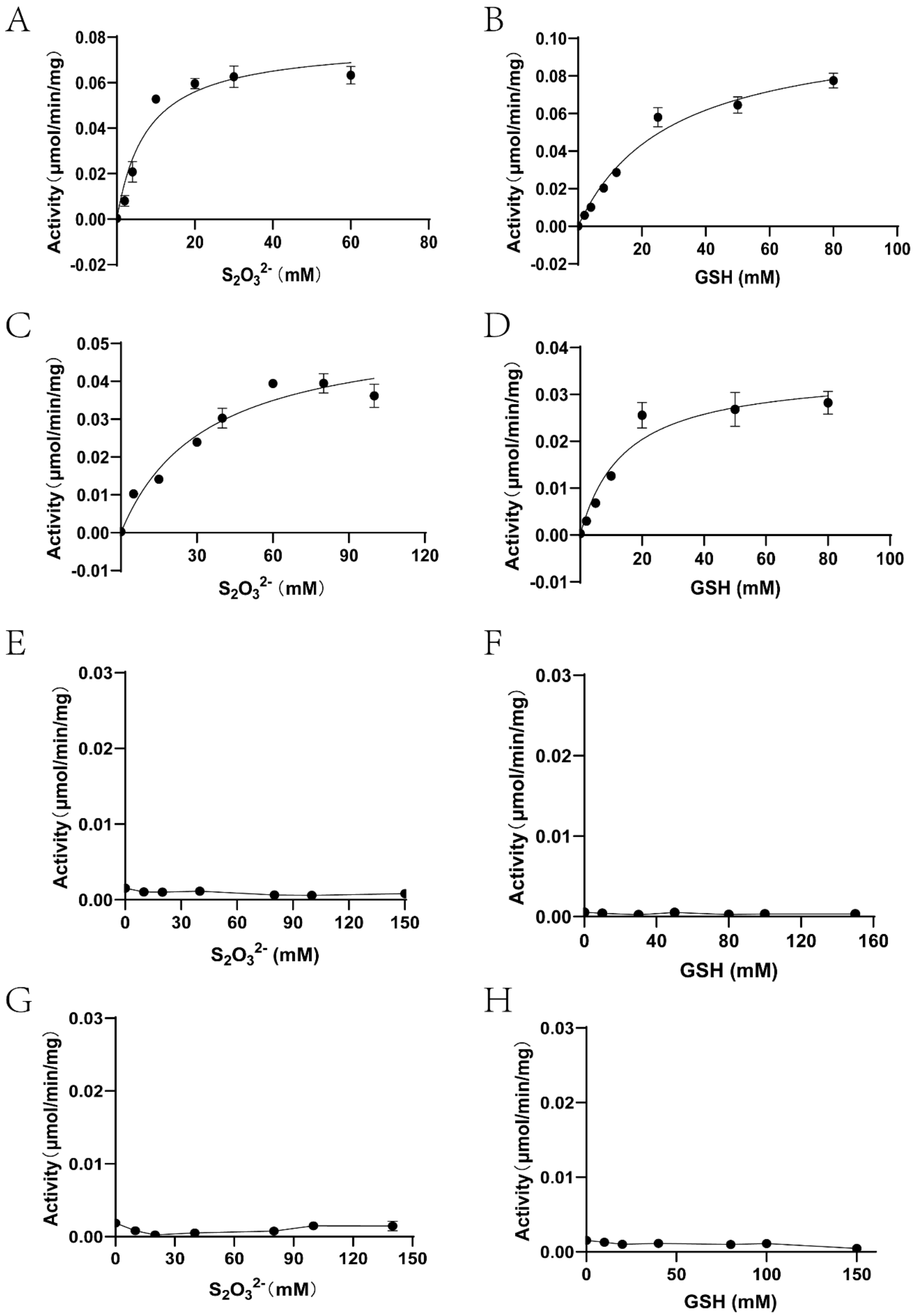

Supplementary Figure S4. Kinetic analysis of RHODs thiosulfate:GSH sulfurtransferase activity. The kinetic parameters of ΔNS-PspE (**A**,**B**), GlpE (**C**,**D**), ΔNS-YnjE (**E**,**F**), and YceA (**G**,**H**) were assayed with either fixed GSH or thiosulfate at 100 mM and varying concentrations of the other substrate. ΔNS-PspE, GlpE, ΔNS-YnjE, and YceA in the reaction

mixtures were at 5 μg/mL, 5 μg/mL, 200 μg/mL, and 100 μg/mL. Three parallel experiments were performed to obtain the averages and standard deviations (n = 3). The data were fitted with Michaelis-Menten equation.

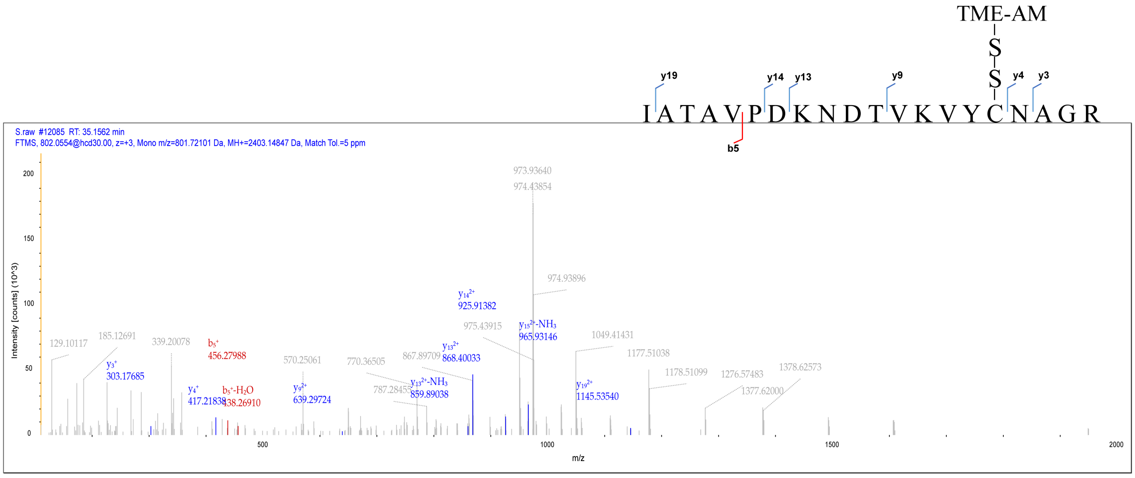

Supplementary Figure S5. LC-MS/MS analysis of Cys49 modification in thiosulfate reacted ΔNS-PspE. Purified ΔNS-PspE protein reacted with thiosulfate for 30 min, sample preparation and LC-MS/MS analysis were then performed. Cys49 persulfidation (Cys-SSH) was identified.

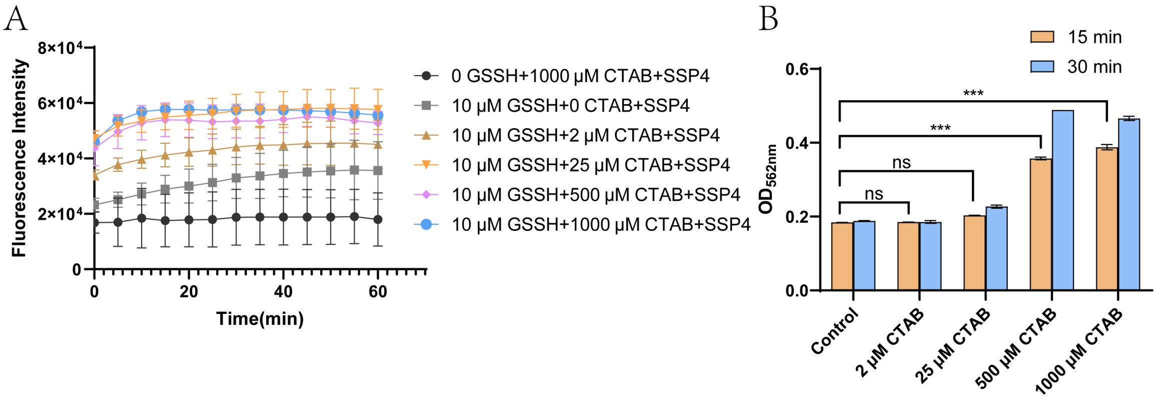

Supplementary Figure S6. Selection of appropriate CTAB concentration. CTAB makes cell membrane more permeable to lead out cellular contents. (**A**) Effects of different concentrations of CTAB on SSP4 fluorescence intensity. (**B**) *E. coli* MG1655 wild-type resting cells at OD_{600nm} of 2.0 were detected with BCA after incubated with different concentrations of CTAB in room temperature. Three parallel experiments were performed to obtain the averages and standard deviations (n = 3).

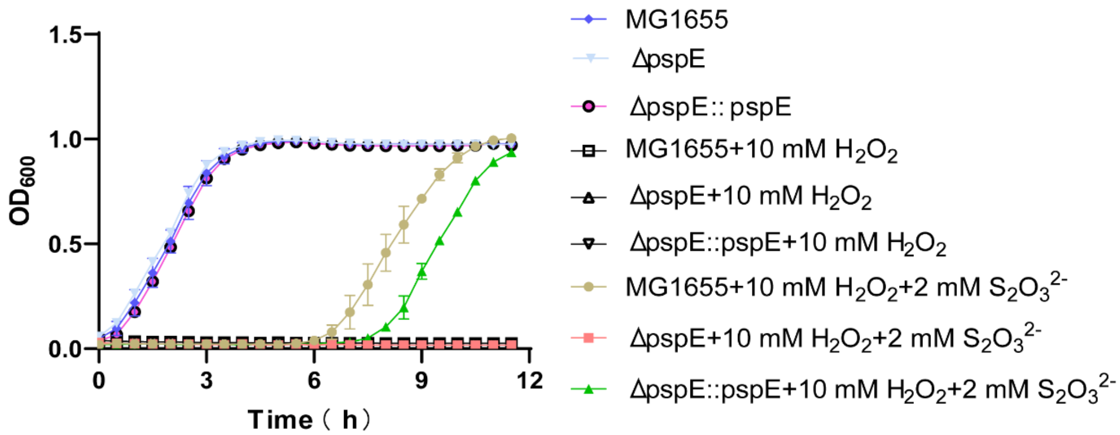

Supplementary Figure S7. Growth curves of *E. coli* when incubated with 2 mM thiosulfate and hydrogen peroxide (H_2O_2). MG1655 wild-type, ΔpspE, and ΔpspE::pspE were incubated in 400 μL LB medium at the initial OD_{600nm} of 0.05 in 48-well plates. No growth was observed for all strains with 10 mM hydrogen peroxide. With 10 mM hydrogen peroxide and 2 mM thiosulfate, delayed growth was observed. However, no growth was observed for ΔpspE with 10 mM hydrogen peroxide and 2 mM thiosulfate. Three parallel experiments were performed to obtain the averages and standard deviations (n = 3).

Supplementary Table S1. Strains and plasmids used in this study

Strain/plasmid	Characteristic	Source
***Escherichia coli* strains**		
DH5α	Cloning strain	Invitrogen
BL21(DE3)	Protein expression strain	Invitrogen
MG1655	Wild type	Laboratory preservation
ΔpspE	MG1655 mutant with *pspE* deleted	Laboratory preservation
ΔygaP	MG1655 mutant with *ygap* deleted	Laboratory preservation
ΔyniE	MG1655 mutant with *ynjE* deleted	Laboratory preservation
ΔglpE	MG1655 mutant with *glpE* deleted	Laboratory preservation
ΔsseA	MG1655 mutant with *sseA* deleted	Laboratory preservation
RHOD-5K	MG1655 mutant with *pspE, glpE, ynjE, ygaP and sseA* genes deleted	Laboratory preservation
RHOD-8K	MG1655 mutant with *pspE, glpE, ynjE, ygaP, sseA, yceA, yibN, ybbB* genes deleted	Laboratory preservation
Plasmids		
pET30a	Km^r, expression vector	Invitrogen
pET30-RHODs	Km^r, pET30a containing ΔNS-*pspE,* ΔNS-*ynjE, glpE, yceA* with C-terminal his-tag	This study
pCP20	pSC101 ori, temperature sensitive, used for antibiotic resistance gene removing	Addgene
pTKred	pSC101 ori, temperature sensitive, used for gene deletion	Addgene
pKD4	R6K ori, Km^r and Amp used for gene deletion or template of pKat promoter	Addgene
pBBR1MCS2-Plac-rhods	Km^r, pMCS2 vector with ΔNS-*pspE*, *pspE, glpE, ynjE, ygaP, sseA, yceA, yibN* or *ybbB* gene from *E. coli* MG1655	This study

Supplementary Table S2. Primers used in this study

Primers	Sequence (5'-3')	Products	Usage
pspE-del-FR	GAAAGTTATGAATACTCGCTGG	1964 bp	For *pspE* deletion
pspE-del-RV	CGCTGTGTAATTAATCGTTTCG		
glpE-del-FR	CCAGCATCAGCACGGATAAACC	2102 bp	For *glpE* deletion
glpE-del-RV	CAATAAACACCACCACGCAGGC		
ynjE-del-FR	TGAGGGAGCTTTCCATCAGGAT	2020 bp	For *ynjE* deletion
ynjE-del-RV	GGCTTTATTCTTGCTGCACCGG		
sseA-del-FR	GCATCTTTTTTCACCGCTGTTG	2007 bp	For *sseA* deletion
sseA-del-RV	AGCAACAAAAAACCGCCTGATT		
ygaP-del-FR	AAAGCAATGAGCCACCCTAAAC	2053 bp	For *ygaP* deletion
ygaP-del-RV	TCAGGGTTGTCACTAAAGAAAG		
pET-ΔNS-PspE-FR	TTAAGAAGGAGATATACATATGGCTGAACACTGGATCGATGTTCG	301 bp	For pET30-ΔNS-PspE construction
pET-ΔNS-PspE-RV	GTGGTGGTGGTGGTGGTGCTCGAGACCTTTGACCTTGGCATTGC		
pET-ΔNS-YnjE-FR	CTTTAAGAAGGAGATATACATATGGCTGAACTGGCGAAGCCTCTTAC	1284 bp	For pET30-ΔNS-YnjE construction
pET-ΔNS-YnjE-RV	GTGGTGGTGGTGGTGGTGCTCGAGTTTGCTACTGTCCGGGCCG		
pET-GlpE-FR	CTTTAAGAAGGAGATATACATATGGATCAGTTCGAATGTATTAACGTTGC	364 bp	For pET30-GlpE construction
pET-GlpE-RV	GGTGGTGGTGGTGCTCGAGCGCGCCGTACGCCAC		
pET-YceA-FR	CTTTAAGAAGGAGATATACATATGCCAGTGTTACACAACCGC	1095 bp	For pET30-YceA construction
pET-YceA-RV	GTGGTGGTGGTGGTGGTGCTCGAGTTCTGTTGGATCAGGAATGCACAG		

Note: PCR was done with Phanta Max Super-Fidelity DNA Polymerase, anneal temperature ranged from 51 to 59 °C, extension was at 72 °C, and denaturing temperature was 95 °C.
